# Neurological Manifestations of Influenza Vaccination: A Narrative Review

**DOI:** 10.7759/cureus.83228

**Published:** 2025-04-29

**Authors:** Hayfa AlAli, Dima Ibrahim, Wdad AlAttar, Moza Almualla, Shahd Ghanim, Fawaghi Robari

**Affiliations:** 1 Department of Primary Healthcare, Emirates Health Services (EHS), Sharjah, ARE; 2 College of Medicine and Health Sciences, Khalifa University, Abu Dhabi, ARE; 3 Department of Infectious Diseases, Burjeel Medical City, Abu Dhabi, ARE

**Keywords:** bell’s palsy, guillain-barré syndrome, influenza, influenza vaccine, narcolepsy, neurologic manifestations, vaccine safety

## Abstract

Influenza poses a significant threat to public health; it remains one of the leading causes of morbidity and mortality worldwide. Vaccination has proven to be the most reliable and effective means of reducing the severity and impact of influenza infection. This review aims to assess the safety profile of the influenza vaccines with a particular focus on potential neurological manifestations. Influenza vaccines are considered generally safe and are recommended for all individuals from the age of six months. However, isolated reports of neurological adverse effects following vaccination have been reported.

In the pediatric population, the main reported manifestations were febrile convulsions and narcolepsy, with the latter primarily associated with the AS03-adjuvanted H1N1 2009 vaccine, Pandemrix. On the other hand, Guillain-Barré Syndrome (GBS) and Bell’s palsy were more commonly reported among the adult population; however, more evidence is needed to support these associations. In conclusion, the overall risk of neurological complications appears to vary depending on the types of vaccine administered. Despite this consideration, the benefits of the influenza vaccine in preventing severe disease and complications significantly outweigh the potential risks. Ongoing surveillance and monitoring are crucial to maintaining influenza vaccine safety.

## Introduction and background

The global burden of influenza is substantial. Annually, influenza infects millions of people worldwide, leading to significant morbidity and mortality. The World Health Organization (WHO) estimates three to five million severe influenza cases, with 290,000 to 650,000 respiratory deaths reported annually [1,2]. Vaccination is the most effective prevention tool against influenza infection and is recommended to be offered to eligible groups during all routine healthcare visits and hospitalizations at any time during influenza season. The Advisory Committee on Immunization Practices (ACIP) advises that individuals aged more than six months without contraindications should receive a seasonal influenza vaccine that is licensed and suitable for their age group [3]. The constant antigenic shifts in the virus pose challenges in vaccine development, necessitating yearly reviews and updates to ensure alignment with the antigenicity of the circulating virus. The history of influenza vaccine development is marked by continuous innovation and improvement. From the initial isolation of the virus in the 1930s to the advanced recombinant DNA techniques, which enable the production of larger quantities of vaccines more efficiently. Furthermore, a new delivery method, the intradermal Fluzone® vaccine, offers superior immunological protection compared to traditional intramuscular vaccines, especially in the elderly. Other notable developments included the introduction of Fluarix®, the first FDA-approved quadrivalent vaccine; FluBlock®, a recombinant trivalent influenza vaccine; and Flucelvax®, the first approved non-egg-produced vaccine alternative. Each milestone has contributed to more effective, high-safety-profile, and widely accessible influenza vaccines. These advancements have played a crucial role in controlling seasonal influenza outbreaks and pandemic preparedness [4,5].

Lately, significant attention has been directed toward crafting a universal vaccine capable of producing broad-spectrum immunity. Three types of vaccines have been approved for the prevention of influenza with varying degrees of immunogenicity (Table 1) [3,6,7]. 

**Table 1:** List of currently available influenza vaccines HA: hemagglutinin; NA: neuraminidase; WPV: whole virus particle vaccine; SV: split viru Adapted from [3,6,7]

Type of vaccine	Sub-types	Age group	Advantages	Disadvantages
Inactivated vaccine	Whole virions/WPV	≥ 6 months	Exhibits higher immunogenicity in animals and humans compared to the split virus vaccine or the subunit vaccine	Abandoned due to its side effects consist of symptoms of an excessive inflammatory response, such as fever, redness, and swelling at the injection site.
	Split-virions/SV	≥ 6 months	The most commonly used vaccines against seasonal influenza.Fewer immunogenic compared with WPV, inducing a moderate immune response with fewer side effects in previously infected or vaccinated individuals.	It requires higher amounts of antigens and the addition of an adjuvant to compensate for the low immunogenicity.
	HA and NA subunit vaccine	≥ 6 months	The most purified vaccine is within the licensed inactivated influenza vaccine. It has weak immunogenicity like the SV, with fewer adverse reactions like fever, compared to WPV.	The lack of immunostimulants in these vaccines leads to low efficacy. Therefore, an adjuvant is added to elderly vaccines (Fluad®) to improve their effectiveness.
Live attenuated vaccine		2 years to 49 years	Mimics a natural infection response. Triggers a strong IgA, IgG response, and cellular immunity (CD4 and CD8 T cell response). Better immunity in young children compared to inactivated vaccines.	Viruses in the vaccine may replicate to a greater extent to a point that might be more harmful than beneficial in those with suboptimal immune systems (pregnancy, immunocompromised, elderly >50 years, children <2 years)
Recombinant vaccine		> 50 years	More efficacious than traditional vaccines by 30% for adults older than 50 years	High price

Influenza vaccines, including inactivated influenza vaccines (IIVs), live attenuated influenza vaccines (LAIVs), and adjuvanted or high-dose formulations used over the past several years, are considered safe for both adults and children. The most common side effect of influenza vaccination remains local irritation at the site of injection and, to a lesser extent, systemic side effects [8, 9]. Although one of the most common causes of influenza-associated hospitalization and death in children following infection includes influenza-associated encephalitis/encephalopathy [10], there were some observations of a possible association between influenza vaccine and neurological manifestations [11-13]. 

The Vaccine Adverse Event Reporting System (VAERS) for the US reports “nervous system disorders” and “general disorders and administration site conditions” as the two most reported categories of non-death serious reports following the administration of inactivated influenza vaccines (IIV4 and IIV3). Two main conditions have been identified under the category of nervous system disorders: Guillain-Barré Syndrome (GBS) in adults (age > 18 years) and febrile seizure in children (age of six months to 17 years) [14]. 

In general, the VAERS database indicates that serious neurological complications following vaccine administration, regardless of vaccine type, are exceedingly uncommon [14]. Therefore, it is complex to draft an association between vaccines and rare diseases like neurological disorders. In this article, we will delve into each suspected association in depth. 

## Review

Guillain-Barré syndrome

GBS is an immune-mediated disorder characterized by an abnormal immune response triggered by an antigenic stimulus, resulting in the immune system attacking self-proteins on peripheral nerves and nerve roots. GBS manifests as a form of polyneuropathy with bilateral ascending paralysis and paresthesia. It is a clinical diagnosis typically preceded by a respiratory or gastrointestinal infection [15]. 

Between October 1, 1976, and January 21, 1977, 1,098 cases of GBS were reported in the United States [16]. Out of 1,098 patients, 532 had recently received an A/New Jersey influenza vaccination before the onset of GBS. Individuals who had been vaccinated were found to be 9.5 times more prone to be diagnosed with GBS compared to those who were unvaccinated (95% CI: 8.2-10.3), consistent with an attributable risk of approximately one case per 100,000 vaccine recipients. Moreover, in this study, the risk of GBS did not differ with vaccine type, manufacturer, or lot, indicating that the matter was unlikely to be related to the manufacturing process. The vaccination program was discontinued before childhood vaccination became widespread, leading to insufficient pediatric cases for meaningful analysis.

Since this initial detection of a link between GBS and the influenza vaccine during the 1976 H1N1 subtype vaccine campaign, numerous studies have been conducted indicating a potential association between GBS and the influenza vaccine [13,17,18]. A significant increase in GBS cases was also observed following vaccination against the influenza A(H1N1)pdm09 virus, with a 57% higher GBS rate than non-vaccinated individuals (the adjusted rate ratio (RR) = 1.57, 95% CI: 1.02, 2.21) [13].

Between 2013 and 2015, cases of GBS were diagnosed and reported following the administration of the quadrivalent inactivated influenza vaccine (IIV4). The symptoms typically emerged around 13 days after vaccination, with a median age of 45 years among affected individuals. Both males and females were affected, with slightly more cases among females [14].

In the 2018-2019 influenza season, a study was done in the United States to search for evidence of any association between the influenza vaccine and GBS in adult patients aged 65 years and above using the Medicare analysis. It was concluded that there was no proven relationship between GBS and the influenza vaccine. It was agreed upon that if such a risk existed, it would be a low risk that is similar in scale to that of prior seasons [18].

In a publication from 2020, 26 articles were analyzed to explore a potential link between GBS and influenza vaccines across all age groups. The review revealed that individuals who received high-dose influenza vaccines faced an elevated risk of developing GBS, with pandemic vaccines posing a greater risk than seasonal ones. Several articles noted a slight increase in the chance of developing GBS regardless of the type of influenza vaccine. However, many studies found no association between the two variables. The lack of a proven association may be attributed to confounding factors such as the simultaneous presence of other influenza-like illnesses, the low weight and power of the studies, and reporting bias in post-vaccination surveillance programs [19].

Even though it was seen more in adults, there were several case reports of GBS in children following the influenza vaccine [20,21]. One of the recent case reports is of a three-year-old female patient who had developed the acute bulbar palsy plus variant of the disease, which occurred around five days after receiving the influenza vaccine. She was treated with IV immunoglobulin, which led to a full recovery in three months [21].

The exact mechanism by which different influenza vaccines may trigger GBS remains unclear. Circulating antibodies are believed to contribute to the pathogenesis of most GBS cases, as manifested by the rapid improvement observed in GBS cases following the administration of high doses of intravenous immune globulin, which is now considered the standard care for GBS. Research has hypothesized that certain antigenic stimuli, such as specific influenza vaccines or influenza viruses, might include cross-reactive antigens that can trigger the autoimmune response observed in GBS [22]. 

In an influenza infection, the autoimmunity mechanism of related GBS involves the secretion of IgG, which binds to the viral surface epitopes and cross-reacts with similar epitopes in the nervous system, leading to nerve cell membrane destruction. This autoimmunity is also proposed as the possible underlying mechanism in vaccine-related GBS [ 23,24].

In conclusion, GBS should be taken into consideration as one of the less common but potentially more serious side effects of an influenza vaccine. Influenza vaccination should be avoided in adults who have experienced GBS within six weeks of a prior influenza vaccination. In such cases, it's advisable to consider chemoprophylaxis using antiviral medications if they have been exposed to the flu. However, for the groups with a higher risk of severe influenza infection complications, the benefit of vaccination might outweigh the risk [3].

Bell’s palsy

Bell’s palsy is the partial or total loss of facial nerve function that can manifest clinically as facial muscle weakness. Facial nerve palsy can occur due to many reasons, including but not limited to infections, trauma, systemic illness, and idiopathic or unknown causes. To a lesser extent, there have been reported cases of Bell’s palsy following certain vaccinations among different age groups. We evaluated certain studies to see whether there was a link between influenza immunization and Bell’s palsy.

Multiple studies have demonstrated a possible temporal association between the influenza vaccine and Bell’s palsy [25-27]. Most studies were in adults; a case-control study looking at Bell’s palsy cases diagnosed between October 2000 and April 2001 in Switzerland found that the relative risk of Bell's palsy was 19 times higher in the vaccinated group compared to the control group, resulting in 13 additional cases per 10,000 vaccinated individuals within 1 to 91 days post-vaccination, with the highest risk period between 31 and 60 days after vaccination. The intranasal inactivated vaccine, which is no longer in clinical use, was predominantly contributing to a higher number of cases compared to parenteral vaccination [25]. In this specific association, the possible mechanism was attributed to vaccine components such as LT toxin, influenza antigens, or the intranasal route of administration [28].

According to the VAERS database, patients who received the influenza vaccination, regardless of the type, had a two-fold increased risk of developing facial palsy compared to those receiving other vaccines, with the highest incidence among 0-14-year-old and 60-74-year-old patients [29].

A study was carried out in Taiwan that consisted of a total of 7,581,205 patients older than 65 years who received the influenza vaccination. The National Health Insurance Research Database (NHIRD) was used to collect patient data retrospectively from the years 2010-2017, and these data were further analyzed in 2022. Older individuals were enrolled due to their increased likelihood of developing more severe infections due to their weakened immune systems. These patients were monitored for six months post vaccination for any signs and symptoms indicating facial nerve palsy. The results showed that a total of 4,367 patients developed Bell’s palsy following the administration of the influenza vaccine. This risk was found to be highest in the first month post-vaccination, more specifically the first week, and the underlying mechanism is still found to be indeterminate [30]. 

On the other hand, a self-controlled case series aimed to see whether there was an association between administering the AS03 adjuvant pandemic H1N1 vaccine and the sequential development of Bell’s palsy in both children and adults. The study population included all diagnosed cases of Bell’s palsy between June 2009 and 2013 in a primary healthcare setting in the UK. The incidence of Bell’s palsy was identified six weeks following vaccination; the study concluded that there was not enough evidence supporting any relation between the two [26].

In the pediatric population, a case-centered analysis concluded that the risk of Bell’s palsy during immunization with a trivalent inactivated vaccine (TIV) was not associated with an increased risk of Bell’s palsy in the first month after immunization [27]. In addition, there have only been a few case reports of Bell’s palsy following influenza vaccination. A case report of a previously healthy seven-month-old infant in 2023 showed that he had developed unilateral Bell’s palsy after receiving an inactivated quadrivalent influenza vaccine. However, his symptoms were fully resolved around 48 hours later, with no episodes of recurrence [31].

The precise pathophysiology of Bell’s palsy following influenza vaccination is uncertain; however, it was generally proposed that an underlying cell-mediated immune response against a myelin protein contributed to a demyelinating neuropathy, particularly in the facial nerve. In addition, the reactivation of a latent virus, possibly due to an immune response triggered by the influenza vaccination, is suggested to play another role in the pathophysiology [32].

In conclusion, there has been conflicting evidence regarding the likelihood of developing Bell’s palsy following the influenza vaccine. After a thorough review, a higher incidence of Bell’s palsy was found in adult age groups compared to the younger population.

Febrile convulsions

Febrile convulsions, or febrile seizures, are the most common seizures in childhood. Febrile convulsions can be triggered by various genetic and environmental factors, including viruses and vaccinations [33].

In a study that examined the safety of the quadrivalent inactivated vaccine (IIV4) through the VAERS in the United States, a total of 512 children were included, and 18 cases of febrile convulsions in children were identified, with 12 of them being verified. The affected children ranged in age from four to 35 months, including 11 males and seven females. The median onset interval from vaccination to febrile seizure onset was 12 hours, ranging from 0 to 12 days. In 14 reports (78%), the IIV4 vaccine was administered alongside other vaccines on the same visit. All 18 children were reportedly recovered [14].

Regarding the trivalent inactivated influenza vaccine (IIV3), a vaccine safety signal for febrile seizure was identified in the 2010-2011 influenza season but not in the subsequent years [34]. In the fall of 2010, an increased risk of febrile seizures was observed in Australia following the administration of the IIV3 vaccine manufactured by Commonwealth Serum Laboratories (CSL) Biotherapies. Surveillance was conducted during the 2010-2011 flu season in a group of 206,174 children aged six to 59 months who received the IIV3 vaccine. This surveillance aimed to detect febrile seizures within 1 day after vaccination. Analysis revealed that the risk of seizures differed based on whether children received the IIV3 vaccine alone or along with the 13-valent pneumococcal conjugate vaccine (PCV13). The risk of seizures was higher when both vaccines were administered together, with the incidence rate ratio (IRR) for concurrent IIV3 and PCV13 being 5.9. The risk was lower for IIV3 or PCV13 alone. The risk also varied by age, with the highest risk for seizures in 16-month-olds and the lowest estimates at 59 months [35]. 

After this association, an observational study in New York was done to evaluate whether children receiving IIV3 and PCV13 simultaneously had higher rates of fever on days 0 to 1 than those receiving either product without the other. The study concluded that administering IIV3 and PCV13 together was linked to a greater temporary increase in fever risk compared to giving either vaccine alone [36]. 

In a systematic review studying fever, febrile convulsions, and serious adverse events following administration of IIV3 in children, the observed rates of febrile convulsions and serious adverse events varied based on the type of study. In randomized controlled trials (RCTs), febrile convulsions occurred at 1.1 per 1,000 vaccinated children. On the other hand, in non-randomized clinical trials, two cases of vaccine-related febrile convulsions were recorded among 2,269 children [37].

While looking at the risk of febrile seizures in children following exposure to pandemic influenza vaccination or infection, a nationwide registry-based study done among the Norwegian population showed that the highest IRR of febrile seizures occurred one to three days after a monovalent pandemic strain vaccine, with an IRR of 2.00. The study concludes that there is an increased risk of emergency hospitalization for febrile seizures following both pandemic influenza vaccination and influenza infection. However, pandemic influenza infection was associated with a greater increase in the risk of febrile seizures [38].

To conclude, the risk of febrile convulsions following influenza vaccination differs based on the age of the recipient, the type of vaccine, and the concurrent use of other vaccines.

Narcolepsy

Narcolepsy is a rare neurological disorder characterized by excessive, disabling daytime sleepiness and occasionally associated with sudden muscle weakness triggered by strong emotions (cataplexy). Narcolepsy, as a possible neurological complication of the influenza vaccine, was a matter of concern after a sudden increase in the incidence of childhood narcolepsy in certain European countries, particularly Finland and Sweden, in 2010 following the use of the AS03-adjuvanted 2009 H1N1 vaccine [11, 39].

Subsequently, many studies were done to investigate the association [40-42]. One notable study was a retrospective analysis done between August 2011 and February 2012 in England, studying the results of sleep tests of children and young adults with narcolepsy. This analysis indicates a causal association with an odds ratio of 16.2 for vaccination six months before the onset of narcolepsy [40].

To reassess the risk of narcolepsy eight years after Pandemrix was introduced, a case-coverage study in England was conducted between September 2017 and June 2018. This study focused on children aged four to 19 years and reported an odds ratio (OR) of 1.94 for narcolepsy onset at any time following AS03-adjuvanted H1N1 subtype pandemic vaccination. The elevated risk period was primarily within 12 months of vaccination, peaking during the first six months. The estimated vaccine-attributable risk was one in 34,500 doses [41]. In a systematic review and meta-analysis conducted in 2017, researchers found that the relative risk of narcolepsy was increased five- to 14-fold in children and adolescents and two- to seven-fold in adults during the first year after vaccination, and this risk was solely limited to the Pandemrix vaccination [43]. Additionally, a comparative analysis in England examined clinically observed differences between vaccinated and unvaccinated children with narcolepsy; cataplexy was the most common initial symptom, reported in 82% of vaccinated patients compared to 55% of unvaccinated patients, though this difference was not statistically significant (p=0.11). However, the study identified a significant difference in the presentation of excessive weight gain as an early clinical feature of narcolepsy, which was more prevalent in vaccinated children (55%) compared to unvaccinated children (20%), with a p-value of 0.03 [42].

While most studies draw the association with the adjuvanted vaccine using ASO3, data on other adjuvanted H1N1 subtype vaccines were limited [44]. Importantly, non-adjuvanted vaccines, such as those used during the 2009 pandemic in the United States, have not been linked with an increased risk of narcolepsy [45].

Several hypotheses have been proposed to explain the pathophysiological mechanism. Narcolepsy is categorized into two main types, narcolepsy type 1 and narcolepsy type 2, with narcolepsy type 1 (NT1) being the focus here. NT1 is likely an immune-mediated destruction of neurons, particularly affecting the endogenous orexin (hypocretin) production in the lateral hypothalamus. This mechanism suggests an autoimmune basis for NT1. While studying narcoleptic patients aged five to 18 years who received the Pandemrix vaccine in 2009 and to understand the mechanism, researchers used an immunofluorescence microscopy assay (IFA) to investigate whether A(H1N1)pdm09 infection or Pandemrix vaccination contributed to the development of autoantibodies targeting the orexin precursor protein or the OX1 or OX2 receptors. No narcolepsy-specific autoantibodies were found in these children. The study concluded that no evidence suggests that the influenza A virus vaccine antigen induces cross-reactive antibodies between the influenza virus nucleoprotein and orexin receptors [46, 47]. More recent research suggests that a T-cell-mediated autoimmune response may play a key role in Pandemrix-associated NT1. In affected children, enhanced T-cell responses were found against influenza viral proteins, as well as a molecular mimic, the brain self-epitope POMT1. Higher levels of anti-POMT1 antibodies were also detected. These findings support an autoimmune mechanism involving T- and B-cell cross-reactivity with POMT1, suggesting it may be a key autoantigen in the development of NT1 [48].

Although the association between the pandemic vaccine and narcolepsy was not considered a worldwide phenomenon, the Finnish National Institute for Health and Welfare (THL), in response to the increased reported cases of narcolepsy, recommended that further Pandemrix vaccinations be discontinued and thorough investigations be conducted. In 2011, THL concluded that there is a clear connection between the Pandemrix vaccination campaign of 2009 and 2010 and the narcolepsy epidemic in Finland, with a four-fold increased risk of narcolepsy in children and adolescents following the Pandemrix vaccine [39,49]. Following these findings, GlaxoSmithKline (GSK), the manufacturer of Pandemrix, ceased its use.

Encephalitis and acute disseminated encephalomyelitis (ADEM)

While numerous cases of encephalitis have been reported in the literature as being potentially triggered by vaccination, including influenza vaccines, no conclusive association has been established [50,51]. A 20-year surveillance study (1990-2010) in the United States identified 1,396 cases of encephalitis following immunization, with influenza vaccines accounting for only 208 cases (14.9%) [52]. Additionally, a case report analyzing MRI findings in four patients who developed neurological conditions after H1N1 influenza vaccination found that encephalitis, although rare, was the most frequently observed complication, along with cerebellitis, neuritis, and myelitis, all of which were associated with MRI abnormalities. Despite these reports, large-scale surveillance studies have not demonstrated a significant increase in encephalitis risk following influenza vaccination. A weekly monitoring study conducted through the Vaccine Safety Datalink (VSD) during the 2012-2013 influenza season found no increased risk of predefined neurological adverse events, including seizures, GBS, encephalitis, or anaphylaxis [53]. Furthermore, an analysis of Norwegian national health registry data from the 2008-2014 influenza seasons found no association between the A(H1N1)pdm09 vaccine and encephalitis, with an adjusted hazard ratio of 0.6 (95% CI: 0.2-2.1) within a 14-day risk window and no reported cases within the first seven days post-vaccination. These findings suggest that while post-vaccination encephalitis has been reported, epidemiological evidence does not support a causal relationship between influenza vaccination and an increased risk of encephalitis.

Regarding ADEM, numerous cases following influenza vaccination have been reported, highlighting the occurrence of this condition post-immunization [54,55]. A nested case-control study examined 272 cases of ADEM and 1,096 matched controls from 2011 to 2015 to evaluate the potential risk of ADEM after vaccination. No elevated risk was identified for various vaccines, including influenza. Furthermore, no association was observed between vaccination and ADEM recurrence. The study concludes that vaccination does not increase the overall risk of ADEM or its recurrence [56]. In 2024, a systematic review examined 23 reported cases of ADEM following influenza vaccination across 19 studies. The average patient age was 40.2 years, with a slight male predominance (60.8%). Common symptoms included muscle weakness (52.1%), urinary abnormalities (30.4%), altered consciousness (26%), and sensory disturbances (26%). Most patients showed full or partial recovery with intravenous steroid treatment, though two (8.6%) died, and a few experienced lasting neurological impairments. The study concludes that while ADEM after influenza vaccination is rare, clinicians should take vaccination history into account in suspected cases, and further research is needed to clarify this potential link [57]. 

**Figure 1:**
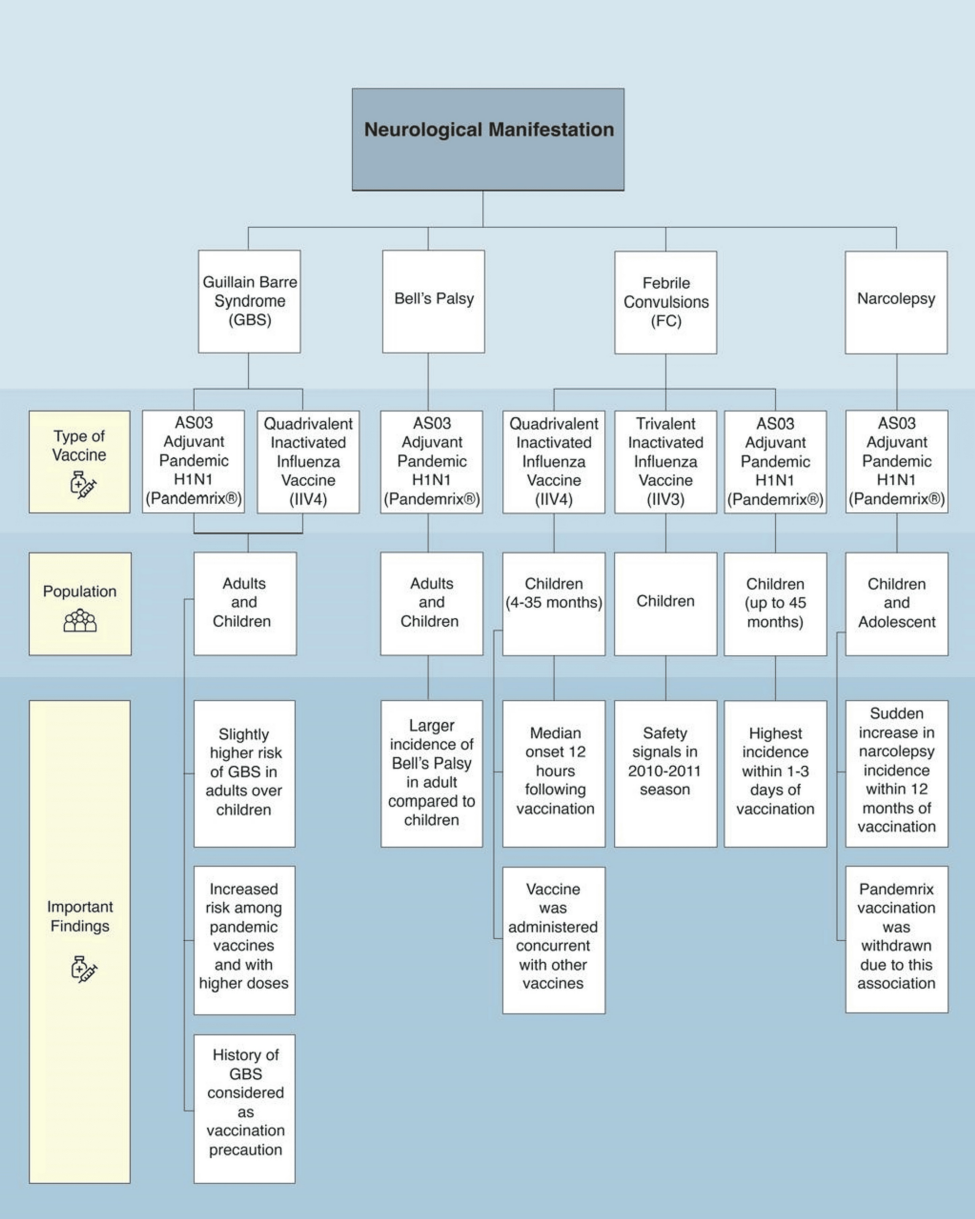
The neurological manifestations of influenza vaccines This flowchart summarizes the neurological adverse events reported in association with different types of influenza vaccines, including Guillain-Barre Syndrome (GBS), Bell’s palsy, febrile convulsions (FC), and narcolepsy. For each condition, the associated vaccine types, affected population groups (adults and/or children), and key findings are outlined. Image credits: Moza Almualla

Figure 1 summarizes the neurologic manifestations of different types of influenza vaccines.

**Figure 2:**
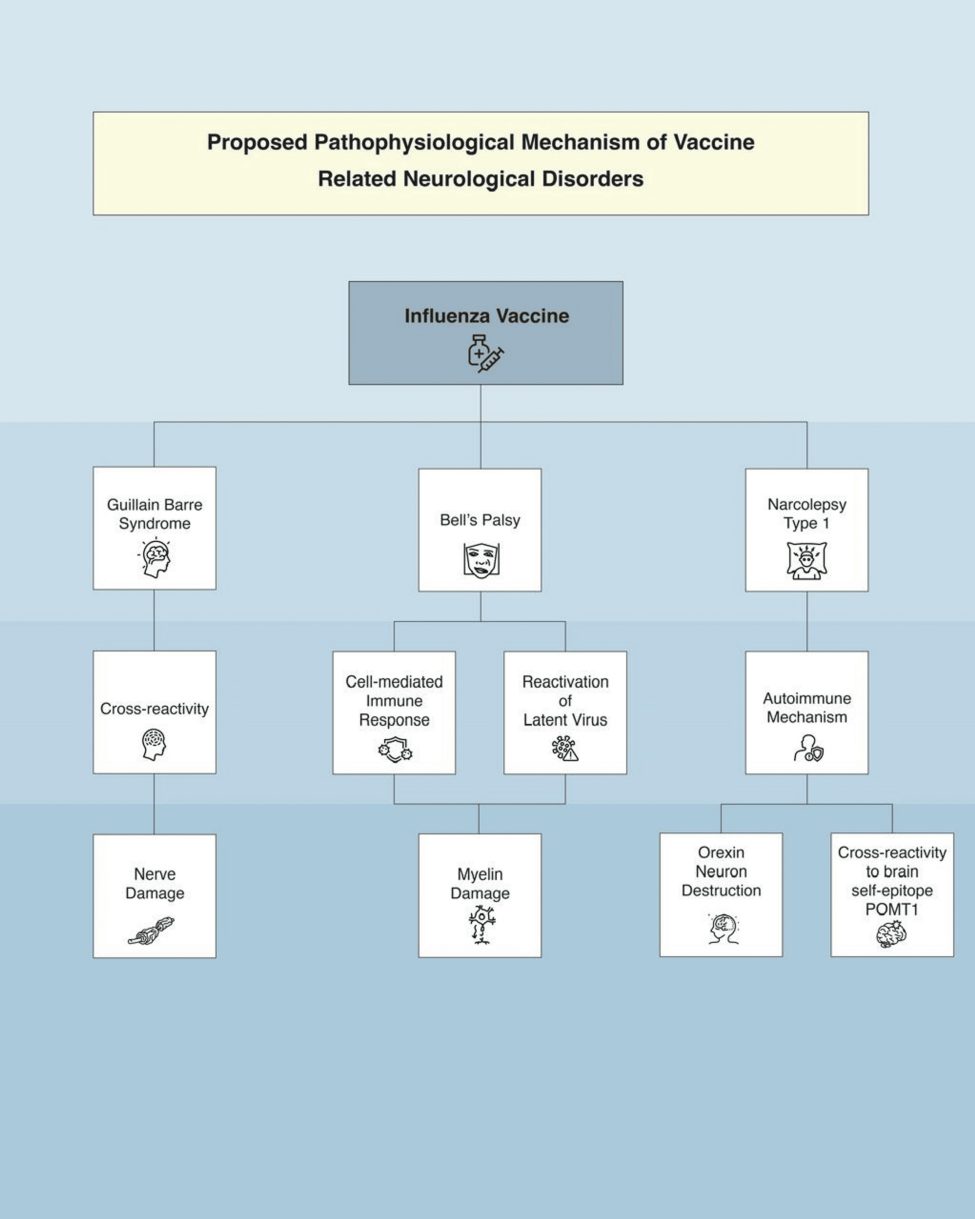
Proposed pathophysiological mechanism of vaccine-related neurological disorders Image credits: Moza Almualla

Figure 2 shows the proposed pathophysiological mechanism of some vaccine-related neurological disorders.

## Conclusions

In conclusion, the influenza vaccinations currently in use have an overall reliable safety record, which encourages their use on an annual basis for all age groups of six months and above. Public health departments are vigilantly monitoring vaccine safety on a global scale to effectively prevent it from causing complications. Nonetheless, we cannot completely rule out the possibility of developing side effects from the vaccine, especially neurological side effects. Although uncertain, the most suggested mechanism of neurological manifestation following influenza vaccines was largely due to an autoimmune response.

One of the significant associations observed was the development of GBS following the influenza vaccination, especially in adults. The available evidence in children is scant and mostly observed in case reports. Nonetheless, even though GBS is not considered a common side effect, it is not to be taken lightly, as it is highly associated with risks and negative outcomes. Therefore, national recommendations signal precautions when vaccinating individuals with a history of GBS six weeks following their previous vaccination. The risk of other neurological complications, such as Bell’s palsy, encephalitis, and ADEM, is not conclusive. The risk of febrile convulsions following influenza vaccination differs based on the types of vaccine and the concurrent use of other vaccines. Moreover, narcolepsy's association with the A(H1N1)pdm09 vaccine was primarily reported in some European countries, such as Finland and Sweden. Despite that, narcolepsy remains a rare side effect of vaccination. In general, it is recommended that researchers continue to explore the immunopathological mechanisms underlying vaccine-associated neurological events, with a particular focus on longitudinal and population-based studies to clarify causality and identify potential risk factors. Vaccine manufacturers should prioritize transparency in safety data reporting, refine vaccine formulations to reduce adverse events, and collaborate closely with regulatory bodies to respond promptly to emerging safety concerns. Additionally, healthcare professionals should consistently communicate the benefits and the rare but potential neurological risks of influenza vaccination to patients and caregivers, adhere to the latest national immunization guidelines, and actively report adverse events to strengthen ongoing pharmacovigilance systems.
